# Indoor Residual Spraying of Insecticide and Malaria Morbidity in a High Transmission Intensity Area of Uganda

**DOI:** 10.1371/journal.pone.0042857

**Published:** 2012-08-06

**Authors:** Ruth Kigozi, Sanjiv M. Baxi, Anne Gasasira, Asadu Sserwanga, Stella Kakeeto, Sussann Nasr, Denis Rubahika, Gunawardena Dissanayake, Moses R. Kamya, Scott Filler, Grant Dorsey

**Affiliations:** 1 Uganda Malaria Surveillance Project, Kampala, Uganda; 2 Department of Internal Medicine, University of California San Francisco, San Francisco, California, United States of America; 3 Malaria Branch, Centers for Disease Control and Prevention, Atlanta, Georgia, United States of America; 4 Uganda Ministry of Health, Kampala, Uganda; 5 President's Malaria Initiative, Kampala, Uganda; 6 Department of Medicine, Makerere University College of Health Sciences, Kampala, Uganda; London School of Hygiene and Tropical Medicine, United Kingdom

## Abstract

**Background:**

Recently the use of indoor residual spraying of insecticide (IRS) has greatly increased in Africa; however, limited data exist on the quantitative impacts of IRS on health outcomes in highly malaria endemic areas.

**Methodology/Principal Findings:**

Routine data were collected on more than 90,000 patient visits at a single health facility over a 56 month period covering five rounds of IRS using three different insecticides. Temporal associations between the timing of IRS and the probability of a patient referred for microscopy having laboratory confirmed malaria were estimated controlling for seasonality and age. Considering patients less than five years of age there was a modest decrease in the odds of malaria following the 1^st^ round of IRS using DDT (OR = 0.76, p<0.001) and the 2^nd^ round using alpha-cypermethrin (OR = 0.83, p = 0.002). Following rounds 3–5 using bendiocarb there was a much greater decrease in the odds of malaria (ORs 0.34, 0.16, 0.17 respectively, p<0.001 for all comparisons). Overall, the impact of IRS was less pronounced among patients 5 years or older.

**Conclusions/Significance:**

IRS was associated with a reduction in malaria morbidity in an area of high transmission intensity in Uganda and the benefits appeared to be greatest after switching to a carbamate class of insecticide.

## Introduction

Malaria is a leading cause of morbidity and mortality in Uganda with an estimated 8–13 million cases per year accounting for 30–50% of outpatient visits, 35% of hospital admissions and 9–14% of hospital deaths, with nearly half of those in children less than 5 years of age [1]. The primary interventions for malaria control in Uganda have been the distribution of insecticide treated bed nets (ITNs) and prompt treatment with artemisinin-based combination therapy (ACT). Historically, indoor residual spraying of insecticide (IRS) has played an important role in the prevention of malaria and more recently, several countries in sub-Saharan Africa have added IRS as part of their malaria control plan in line with the *Global Malaria Action Plan* launched by the WHO-Roll Back Malaria Partnership [2]. In 2006, Uganda began using IRS in selected districts, initially focusing on epidemic prone regions in the Southwest, but subsequently shifting to highly endemic areas in the North in accordance with the WHO position statement on IRS in hyperendemic areas [3].

IRS was a critical component of the WHO's Global Malaria Eradication Program from 1955–1969 and the main intervention contributing to the elimination or dramatic reduction of malaria in parts of Europe, Asia, and Latin America [4]. Additionally, several historical reviews have documented programmatic benefits of successful IRS programs in Africa, primarily in the southern part of the continent [5], [6], [7], [8], [9], [10]. These reviews reported clear and substantial reductions in various malaria related outcomes and measures of vector density, and in some instances local elimination was achieved. However, most of these studies lacked an appropriate control group needed to accurately quantify the impact of IRS. A recent Cochrane Review of IRS for preventing malaria using strict criteria necessary to quantify impact identified only 4 studies from Africa, of which 3 were from areas of stable transmission and 2 were randomized controlled trials [4]. Thus, there is an urgent need for additional studies aimed at estimating the quantitative impact of IRS on relevant health outcomes in different epidemiological settings. In addition, resistance to insecticides including dichloro-diphenyl-trichloroethane (DDT) and several pyrethroids have become of increasing concern in Uganda and other parts of Africa [11], [12], [13], [14], [15]. The WHO recommends a number of insecticides for IRS, however, there is limited evidence to guide which class of insecticide should be used in different settings.

In this study, data from a health facility-based malaria surveillance program were used to estimate the impact of IRS on malaria morbidity in the Apac District of Uganda, an area of very high transmission intensity. The probability of having malaria parasitemia among patients referred for microscopy from the outpatient department of a single government health center was used as the primary indicator of morbidity. These data were collected over a 4 ½ year period, covering 5 rounds of IRS using 3 different classes of insecticide.

## Methods

### Study site and participants

The Ugandan Malaria Surveillance Program (UMSP) in collaboration with the National Malaria Control Program (NMCP) runs a sentinel site health facility-based malaria surveillance system at six level IV government health centers around the country. As part of this program individual-level data on all patients presenting to the outpatient department of the sentinel site facilities are collected using an electronic data collection system. Data collected includes patient demographic information, basic clinical information, laboratory results, diagnoses, and treatments prescribed. A strong emphasis is placed on referral of patients with suspected malaria for laboratory testing using microscopy (thick blood smear) and the training of health care workers and laboratory technicians [16].

This study included one sentinel site health facility, Aduku Health Center, located in the Apac district of Northern Uganda. Malaria transmission in this area is perennial, with 2 peaks following the rainy seasons, and had an entomological inoculation rate estimated to be 1586 infectious bites per person per year in 2001 [17]. Of note, the activities of this sentinel site malaria surveillance program were established independent of the IRS activities described below.

### Ethics statement

The data included in this report were considered routine public health surveillance and not classified as research requiring approval from an institutional review board according to CDC policy (http://www.cdc.gov/od/science/integrity/docs/cdc-policy-distinguishing-public-health-research-nonresearch.pdf). Data did not include any patient identifiers and were analyzed anonymously.

### Description of IRS activities in the Apac District

Over the 4 ½ year study period, 5 rounds of IRS were completed (Table 1). Round 1 occurred from March-May 2008 using DDT, round 2 occurred from March-April 2010 using the pyrethroid alpha-cypermethrin, and rounds 3–5 occurred at approximately 5 months intervals from August 2010 through June 2011 using the carbamate bendiocarb. Coverage of households sprayed and population protected was over 90% in round 1 and over 99% in rounds 2–5. The almost 2 year delay between the 1^st^ and 2^nd^ rounds of IRS was due to a court injunction by local organic farmers against the use of DDT. The switch from a pyrethroid to a carbamate class of insecticide between the 2^nd^ and 3^rd^ rounds was based on evidence demonstrating significant resistance to both DDT and pyrethroids but no resistance to carbamates among *Anopheles gambiae* in the area (submitted for publication).

**Table 1:** Details of indoor residual insecticide spraying (IRS).

Formulation of insecticide	Dates of spraying	Percentage of households sprayed	Percentage of the population protected
DDT	March 2008–May 2008	92.4%	91.0%
alpha-cypermethrin	March 9^th^ 2010–March 31^st^ 2010	99.9%	99.9%
bendiocarb	August 23^rd^ 2010–September 21^st^ 2010	99.6%	99.6%
bendiocarb	January 5^th^ 2011–January 29^th^ 2011	99.4%	99.3%
bendiocarb	May 23^rd^ 2011–June 20^th^ 2011	96.8%	96.8%

### Statistical analysis

This study utilized data collected as part of the sentinel site malaria surveillance system from March 2007 through October 2011. Suspected malaria was defined as all patients referred for malaria laboratory testing plus all patients not referred to the laboratory but given a clinical diagnosis of malaria. The primary exposure variable of interest was calendar time in relationship to IRS. A categorical time variable was created that included the 6 month period following the 1^st^ round of IRS with DDT, each 4 month period following rounds 2–5 of IRS with α-cypermethrin and then bendiocarb. The longer period following DDT was done to reflect its longer duration of action and the fact that the period between rounds 2–5 was approximately 4 months. The baseline time period (for which each post-IRS time period was compared with) included all observations between March 2007 through October 2011 excluding the time periods of interest following each round of IRS described above. The primary outcome of interest was the proportion of patients with blood slide results (denominator) that were positive for asexual parasites (numerator), commonly referred to as the slide positivity rate (SPR). Associations between time periods of interest and the probability of having a positive blood slide result if a blood slide result was done were expressed as an odds ratio (OR) using a logistic regression model controlling for age, monthly seasonality and autocorrelation by including a quadratic term for the day of observation. Data were stratified for patients under 5 years of age and those 5 years of age or older. Analyses were performed separately for all patients regardless of residence and then restricted to only those who reported residing within the Aduku sub-county where the sentinel site health center was located. A p-value <0.05 was considered statistically significant.

## Results

### Characteristics of patients

Characteristics of individual patient level data collected as part of the surveillance system are included in Table 2. In total, 90,231 patient encounters were included over the 56 month study period. Overall 51% of patients had suspected malaria. Interestingly, the proportion of patients with suspected malaria increased significantly between the first and second half of the observation period (44% vs. 57%, p<0.001). Among patients with suspected malaria less than 1% had no age recorded and 16% had no blood smear results. The proportion of patients with suspected malaria who did not undergo microscopy decreased significantly from 37% during the first half of the observation period to less than 3% during the second half of the observation period (p<0.001). A total of 38,336 patient encounters among patients with suspected malaria, reported age, and microscopy results were included in the analyses of temporal changes in morbidity in relationship to IRS. Among patients with complete data, less than 1% reported residing outside the Apac district where IRS was conducted and 57% reported residing in the Aduku sub-county where the sentinel site health facility was located.

**Table 2:** Characteristics of the study population.

	Entire observation period (March 2007–October 2011)	First half of observation period (March 2007–June 2009)	Second half of observation period (July 2009–October 2011)
Total number of patients encounters	90,231	42,619	47,612
Suspected malaria (% total)	46,090 (51%)	18,944 (44%)	27,146 (57%)
Missing data if malaria suspected			
No age	120 (0.3%)	42 (0.2%)	78 (0.3%)
No blood smear result	7,734 (16%)	6,930 (37%)	704 (2.6%)
Complete data if malaria suspected	38,336 (83%)	11,972 (63%)	26,364 (97%)
Geographic strata if malaria suspected and complete data			
Residence missing	5,252 (14%)	4,016 (34%)	1,236 (4.7%)
Resident outside Apac district	95 (0.3%)	45 (0.4%)	50 (0.2%)
Resident of Apac district outside Aduku sub-county	11,236 (29%)	2,479 (21%)	8,757 (33%)
Resident of Aduku sub-county	21,753 (57%)	5,432 (45%)	16,321 (62%)
Age strata if malaria suspected and complete data			
<5 years of age	16,382 (43%)	6,452 (54%)	9,930 (38%)
≥5 years of age	21,954 (57%)	5,520 (46%)	16,434 (62%)

### Associations between the timing of IRS and temporal changes in malaria morbidity

**Figure 1:**
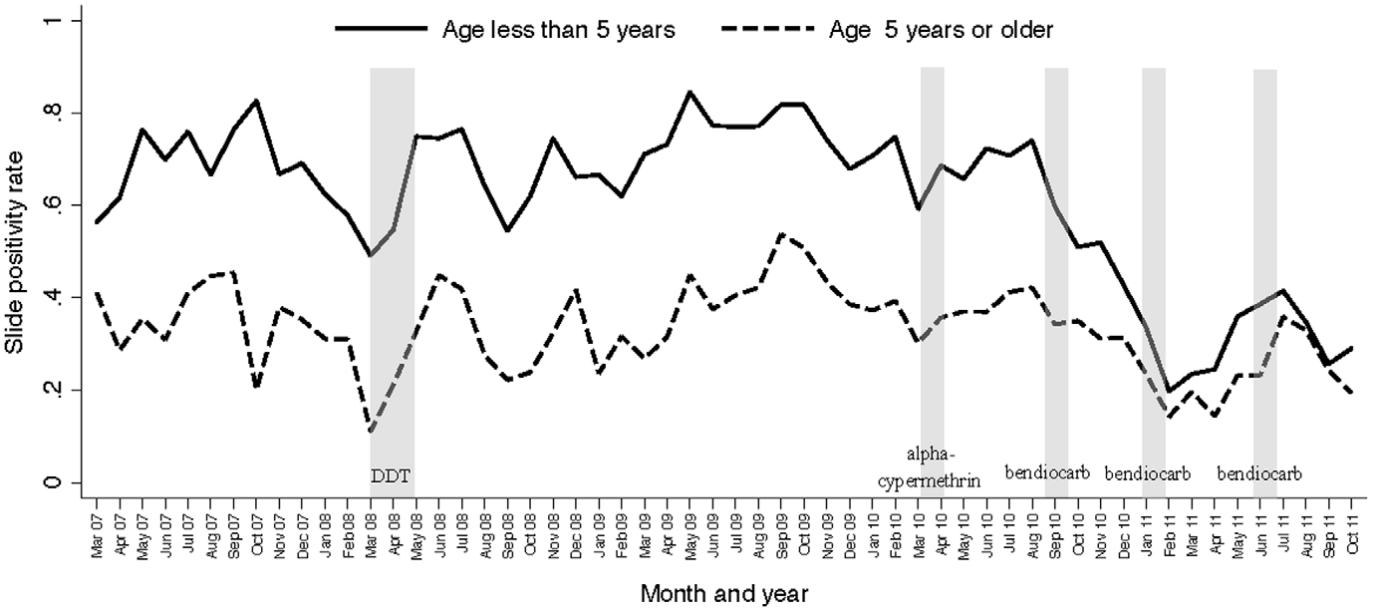
Monthly trends in malaria slide positivity stratified by age groups among patients with suspected malaria referred for laboratory testing at the Aduku Health Center in Apac, Uganda. Vertical bars represent the duration of each round of IRS.

Temporal trends in the monthly SPR stratified by age groups are presented in Figure 1. Considering the reference time period, the SPR was 71% for patients under 5 years of age and 36% for patient 5 years of age or older. During the 6 months following the 1st round of IRS with DDT there was a significant decrease in the odds of having malaria among patients less than 5 years of age (OR = 0.76, 95% CI 0.67–0.86, p<0.001) and those 5 years of age or older (OR = 0.70, 95% CI 0.61–0.79, p<0.001). During the 4 months following the 2nd round of IRS with alpha-cypermethrin there was a significant decrease in the odds of having malaria among patients less than 5 years of age (OR = 0.83, 95% CI 0.73–0.93. p = 0.002), but not among those 5 years of age or older (OR = 1.09, 95% CI 0.99–1.22. p = 0.10) (Table 3). During the 4 months following the 3rd round of IRS with bendiocarb there was a significant decrease in the odds of having malaria that was more pronounced among patients less than 5 years of age (OR = 0.34, 95% CI 0.30–0.40, p<0.001) compared to those 5 years of age or older (OR = 0.76, 95% CI 0.68–0.84, p<0.001)(p<0.001 for interaction when comparing effects between two age groups). The greatest decrease in the odds of having malaria occurred during the 4 months following the 4^th^ and 5^th^ rounds of IRS using bendiocarb and was more pronounced among patients less than 5 years of age (ORs 0.16, 95% CI 0.14–0.19, p<0.001; and 0.17, 95% CI 0.15–0.20, p<0.001) compared to those 5 years of age or older (ORs 0.44, 95% CI 0.38–0.50, p<0.001, and 0.58, 95% CI 0.52–0.65, p<0.001)(p<0.001 for interaction when comparing effects between two age groups). There was a sharp decline in the SPR following the 3^rd^ round of IRS (1^st^ round with bendiocarb), sustained initially after the 4^th^ round of IRS, then increasing prior to the 5^th^ round followed by another sharp decline (Figure 1). Results were similar when restricting the analyses to only patients who reported residing in the Aduku sub-county (Table 3).

**Table 3:** Associations between the timing of IRS and temporal changes in malaria morbidity.

Timing in relationship to IRS	Including all patients							
	<5 years of age				≥5 years of age			
	OR* (95% CI)	p-value	OR† (95% CI)	p-value	OR* (95% CI)	p-value	OR† (95% CI)	p-value
Baseline‡	1.0 (ref)	-	1.0 (ref)	-	1.0 (ref)	-	1.0 (ref)	-
6 months after completion of 1^st^ round with DDT	0.91 (0.82–1.02)	0.11	0.76 (0.67–0.86)	<0.001	0.82 (0.73–0.93)	0.001	0.70 (0.61–0.79)	<0.001
4 months after completion of 2^nd^ round with alpha-cypermethrin	0.96 (0.86–1.07)	0.48	0.83 (0.73–0.93)	0.002	1.12 (1.03–1.23)	0.01	1.09 (0.98–1.22)	0.10
4 months after completion of 3^rd^ round with bendiocarb	0.36 (0.31–0.41)	<0.001	0.34 (0.30–0.40)	<0.001	0.79 (0.72–0.86)	<0.001	0.76 (0.68–0.84)	<0.001
4 months after completion of 4^th^ round with bendiocarb	0.14 (0.12–0.17)	<0.001	0.16 (0.14–0.19)	<0.001	0.40 (0.36–0.44)	<0.001	0.44 (0.38–0.50)	<0.001
4 months after completion of 5^th^ round with bendiocarb	0.21 (0.18–0.24)	<0.001	0.17 (0.15–0.20)	<0.001	0.68 (0.62–0.75)	<0.001	0.58 (0.52–0.65)	<0.001

Timing in relationship to IRS	Including only patients living in Aduku sub-county							
	<5 years of age				≥5 years of age			
	OR* (95% CI)	p-value	OR† (95% CI)	p-value	OR* (95% CI)	p-value	OR† (95% CI)	p-value
Baseline‡	1.0 (ref)	-	1.0 (ref)	-	1.0 (ref)	-	1.0 (ref)	-
6 months after completion of 1^st^ round with DDT	0.89 (0.77–1.04)	0.14	0.76 (0.64–0.89)	0.001	0.78 (0.66–0.91)	0.002	0.63 (0.53–0.75)	<0.001
4 months after completion of 2^nd^ round with alpha-cypermethrin	0.90 (0.78–1.03)	014	0.76 (0.65–0.90)	0.001	1.12 (0.99–1.26)	0.06	1.12 (0.98–1.29)	0.11
4 months after completion of 3^rd^ round with bendiocarb	0.32 (0.27–0.37)	<0.001	0.31 (0.26–0.38)	<0.001	0.80 (0.71–0.90)	<0.001	0.76 (0.67–0.88)	<0.001
4 months after completion of 4^th^ round with bendiocarb	0.13 (0.11–0.16)	<0.001	0.14 (0.11–0.17)	<0.001	0.39 (0.34–0.44)	<0.001	0.43 (0.36–0.51)	<0.001
4 months after completion of 5^th^ round with bendiocarb	0.20 (0.17–0.24)	<0.001	0.16 (0.13–0.19)	<0.001	0.65 (0.57–0.73)	<0.001	0.53 (0.46–0.61)	<0.001

* Unadjusted odds ratio for having malaria among patients referred for microscopy.

† Adjusted odds ratio for having malaria among patients referred for microscopy controlling for age, seasonality and autocorrelation.

‡ Includes all observations between March 2007 through October 2011 excluding the time periods of interest following each round of IRS.

Over the course of the IRS program there was a demographic shift to an older age range among patients with suspected malaria referred for microscopy. The proportion of patients referred for microscopy who were under 5 years of age was 32% and remained relatively unchanged before the first round of IRS through the period 4 months after the 2^nd^ round of IRS. However, in the 4 months following the 3^rd^ through 5^th^ rounds of IRS, the proportion of patients referred for microscopy who were under 5 years of age decreased to 19% (p<0.001).

## Discussion

The Uganda National Malaria Control Program (NMCP) strategic plan now recognizes IRS as one of the major malaria prevention and control interventions in the country. The NMCP began large-scale IRS campaigns in selected districts with support from the U.S. President's Malaria Initiative in 2006 after an absence of such activities since the 1960s. Since 2008, the IRS program has focused on ten high transmission districts in Northern Uganda, targeting nearly 3 million people living in 850,000 households. Due to emerging vector resistance patterns the choice of insecticide class has evolved from DDT to pyrethroids to carbamates. Vector resistance monitoring using standard WHO testing methods conducted from August-October 2009 in this area revealed *Anopheles gambiae* was highly resistant to DDT, partially resistant to pyrethoids (etofenprox and lamdacyhalothrin), and full susceptibility to both carbamates and organophosphates (submitted for publication). Here we describe the first published report of the impact of IRS on health outcomes in one of these high transmission districts of Uganda (Apac) using a health-facility based malaria surveillance system. In the 6 months following the first round of IRS with DDT there was evidence of a modest decrease in measures of malaria morbidity. Following a 22 month delay between the 1^st^ and 2^nd^ rounds of IRS, there was again evidence of modest decrease in malaria morbidity in the 4 months following the 2^nd^ round of IRS with alpha-cypermethrin, but only among patients less than 5 years of age. Following the 3^rd^ through 5^th^ rounds of IRS with bendiocarb, there was a more dramatic decrease in malaria morbidity. These decreases were greatest in the younger age group and there was a shift towards an older patient population presenting with suspected malaria.

The use of IRS in the African region has greatly increased in recent years with 36 countries recommending IRS and an estimated 78 million people protected in 2010, up from 10 million in 2005 [18]. Despite the dramatic recent increase in the use of IRS and clear evidence from historical reviews in Africa that IRS reduces transmission and leads to substantial epidemiological benefit, there are very limited data from controlled trials quantifying the impact of IRS on health outcomes from this region [4]. In a randomized controlled trial (RCT) of IRS with the pyrethroid deltamethrin versus ITNs in an area of unstable transmission in South Africa, IRS was associated with a 34% protective efficacy in the incidence of infection, but this difference was not statistically significant [19]. In a RCT from a highly endemic area of Tanzania, the use of IRS with the pyrethroid lambdacyhalothrin compared to a control group with no intervention was associated with a 54% reduction (95% CI 49–58%) in the incidence of reinfection and a 14% reduction (95% CI 5–23%) in the incidence of malaria among children 1–5 years but no difference in parasite prevalence or the incidence of malaria among children older than 5 years [20]. In a controlled before-and-after study from the early 1970's in an area of stable malaria transmission in Nigeria, the use of IRS with the carbamate propoxur was associated with a 26% protective efficacy (95% CI 20–32%) against malaria prevalence during the wet season but no protection during the dry season [21]. In an interrupted time series study from an area of stable transmission in Mozambique, sustained use of IRS with the carbamate bendiocarb over a 5 year period was associated with a 74% protective efficacy (95% CI 72–76%) against malaria prevalence [22]. Finally, using health facility-based data from an area of moderate transmission intensity in Southwest Uganda, a single round of IRS with the pyrethroid lambdacyhalothrin was associated with a decrease in the SPR from 47% to 14% among patient less than 5 years of age during the 4 month period following the intervention but this effect waned over the subsequent 12 months [23]. The results presented in this study provide further evidence of the quantitative impact of IRS on measures of malaria morbidity in an area of high transmission intensity and is novel in its assessment of multiple rounds of IRS using different classes of insecticide. One of the interesting findings in this study was the more profound decrease in malaria morbidity following IRS with bendiocarb in patient under 5 years of age compared to those 5 years of age or older. This difference may be due to lower levels of antimalarial immunity in younger patients, making them more dependent on interventions for the control of malaria.

The choice of class of insecticide for IRS has become a critical issue with the emergence of insecticide resistance. In 2009, pyrethroids were estimated to account for 77% of spray area covered, DDT accounted for 20% of sprayed areas, and carbamates and organophosphates represented a very small proportion of global usage for vector control [18]. However, recent data suggest that resistance to DDT and various pyrethroids among the main *Anopheles* vectors may be commonplace in many parts of Africa, including Uganda [18]. The main vector species in Uganda are *An. gambiae* and *An. funestus* [*17*] and resistance to DDT and pyrethoids have been described recently to both of these species in the central and eastern part of the country [13], [14], [15]. In the Apac district, the site of this study, data collected from 2004–06 demonstrated significant resistance by *An. gambiae* to DDT (24-hr mortality 63–76%) and permethrin (24-hr mortality 80–81%) and to a lesser extent by *An. funestus* to DDT (24-hr mortality 81–100%) and permethrin (24-hr mortality 92–99%) [15]. Similar patterns of insecticide resistance have been reported in other African countries [24] and a recent report from Benin documented dramatic decreases in entomological measures of transmission following large-scale IRS with bendiocarb in areas with high resistance of *An. gambiae* to pyrethroids [25]. However, this is the first report documenting substantial decreases in measures of malaria morbidity following repeated rounds of IRS with bendiocarb after more modest decreases following IRS with DDT and then pyrethroids.

There were several limitations to this study. An interrupted time series study design was used therefore caution should be taken when making causal inferences about the quantitative impact of the intervention given the lack of a contemporaneous control group. Temporal changes in measures of malaria morbidity could have been influenced by several potential confounding factors which were not controlled for such as environmental factors associated with malaria transmission intensity and changes in the coverage of other malaria control interventions. For example, changes in the use of ITNs were not controlled for in this study. Data from malaria indicator surveys done in the mid-Northern region of the country involving 9 districts including Apac reported 64% of households owned at least one ITN in 2009 and this increased to 82% in late 2010 (Uganda Ministry of Health, unpublished data). Therefore decreases in measures of malaria morbidity seen in this study could have been partially due to increasing ITN coverage and not just a result of IRS. In a recent non-randomized prospective cohort study from Western Kenya, IRS with ITNs was associated with a 62% protective efficacy against the incidence of malaria parasitemia compared to ITNs alone [26]. This study is also unable to separate out the independent effects of the various rounds of IRS using different classes of insecticide. For example the dramatic decrease in malaria morbidity seen after rounds 3–5 using bendiocarb may have been different if this was not preceded by the previous 2 rounds using DDT and alpha-cypermethrin, respectively. Indeed, it has been suggested that in very high transmission settings several years of IRS may be needed to have an appreciable health impact [27]. Another limitation of this study is the use of the SPR as the primary outcome measure of malaria morbidity. The SPR has a non-linear relationship with malaria incidence in a target population and may be influenced by other factors such as the incidence of non-malarial fevers [28]. In addition, the SPR is dependent on which patients are determined to have suspected malaria and the proportion of patients with suspected malaria who undergo diagnostic testing. Both of these factors changed over the course of the observation period and could have led to bias in estimates of SPR, although this potential source of bias could have changed estimates of SPR in either direction. Despite these limitations, the use of health facility-based data remains an efficient and practical means of monitoring temporal changes in malaria morbidity in the setting of routine malaria surveillance. Despite these limitations, the consistent and profound decrease in SPR seen following the last three rounds of IRS in this study provides evidence for the overall effectiveness of a sustained IRS program in a high transmission setting on reducing the burden of malaria, although caution must be taken about making causal inferences about the temporal associations between IRS and malaria morbidity given the study design.

In summary, IRS was associated with a reduction in malaria morbidity in an area of high transmission intensity in Uganda and the benefits appeared to be greatest after switching to a carbamate class of insecticide. Further studies are needed to better quantify the impact of IRS in different epidemiological settings and identify the optimal class of insecticides and spraying schedules. In addition, future studies should also attempt to better delineate the incremental benefits of IRS in the setting of other malaria control interventions.
